# Effects of Electroacupuncture at Auricular Concha Region on the Depressive Status of Unpredictable Chronic Mild Stress Rat Models

**DOI:** 10.1155/2013/789674

**Published:** 2013-01-29

**Authors:** Ru-Peng Liu, Ji-Liang Fang, Pei-Jing Rong, Yufeng Zhao, Hong Meng, Hui Ben, Liang Li, Zhan-Xia Huang, Xia Li, Ying-Ge Ma, Bing Zhu

**Affiliations:** ^1^Institute of Acupuncture and Moxibustion, China Academy of Chinese Medical Sciences, Beijing 100700, China; ^2^Guanganmen Hospital, China Academy of Chinese Medical Sciences, Beijing 100053, China; ^3^Clinical Evaluation Center, Institute of Basic Research in Clinical Medicine, China Academy of Chinese Medical Sciences, Beijing 100700, China; ^4^Beijing University of Chinese Medicine, Beijing 100029, China

## Abstract

To explore new noninvasive treatment options for depression, this study investigated the effects of electroacupuncture (EA) at the auricular concha region (ACR) of depression rat models. Depression in rats was induced by unpredictable chronic mild stress (UCMS) combined with isolation for 21 days. Eighty male Wistar rats were randomly assigned into four groups: normal, UCMS alone, UCMS with EA-ACR treatment, and UCMS with EA-ear-tip treatment. Rats under inhaled anesthesia were treated once daily for 14 days. The results showed that blood pressure and heart rate were significantly reduced in the EA-ACR group than in the UCMS alone group or the EA-ear-tip group. The open-field test scores significantly decreased in the UCMS alone and EA-ear-tip groups but not in the EA-ACR group. Both EA treatments downregulated levels of plasma cortisol and ACTH in UCMS rats back to normal levels. The present study suggested that EA-ACR can elicit similar cardioinhibitory effects as vagus nerve stimulation (VNS), and EA-ACR significantly antagonized UCMS-induced depressive status in UCMS rats. The antidepressant effect of EA-ACR is possibly mediated via the normalization of the hypothalamic-pituitary-adrenal (HPA) axis hyperactivity.

## 1. Introduction

Vagus nerve stimulation (VNS) was approved by the U. S. Food and Drug Administration in 2005 and has been frequently used as a treatment option for treatment-resistant depression (TRD) [1–4]. Its mechanisms of antidepressant action are not fully elucidated; however, its neuromechanisms are based on the direct stimulation of the cervical trunk of the left vagus nerve. The afferent fibers of vagus nerve project to solitary nucleus (SN). Fibers of SN project to the neuroendocrine systems in the limbic system structures and the autonomic nervous system. These areas are strongly interconnected by monoamine-related pathways, including the ventral tegmental area, brainstem, the hypothalamus, thalamus, amygdala, anterior insula, nucleus accumbens, and the lateral prefrontal cortex [5]. Furthermore, the ventral tegmental area has a dense dopaminergic input to the prefrontal cortex; fibers from the SN project to the locus ceruleus and dorsal raphe nucleus which are major brainstem nuclei related to noradrenergic (NE) and serotonergic (5-HT) innervations of the entire brain cortex, respectively. It is well known that the serotonergic, dopaminergic, and noradrenergic systems are commonly involved in the pathophysiology of depression and in the neuromechanisms of action of antidepressants [6].

Nonetheless, typical implantation of VNS device requires an invasive surgical procedure which may be accompanied by some side effects, such as infection of wound, hoarse voice, dyspnea, difficulty swallowing, neck pain, paresthesia, emesis, laryngospasms, dyspepsia, cardiac asystole, bradycardia, and even heart failure. Worse still, technical complications of device malfunction may aggravate patient conditions [7, 8].

Enlightened by the mechanism of VNS, researchers in our team found that auricular concha region (ACR) is densely innervated by free nerve endings of the vagus nerve. Our previous animal studies found that electroacupuncture (EA) at ACR (EA-ACR) had significant effects in the management of primary hypertension [9, 10], diabetes mellitus [11, 12], and partial epilepsy [13, 14]. EA-ACR is a noninvasive procedure which requires a portable EA device and no side effects. With a similar mechanism to VNS, EA vagus nerve stimulation may provide beneficial effects in the treatment of depression.

EA-ACR is one of the acupuncture therapeutic methods which can be considered as auriculotherapy. Theories of auriculotherapy dates back to 2000 years ago as first mentioned in the book of *Yellow Emperor's Canon of Medicine* (Huang Di Nei Jing) [15]. Modern auriculotherapy with 42 points was firstly introduced by Dr. P. Nogier (France) in 1956. The international standard map of auricularpoints was published in China and later was recommended by WHO in 1993. Acupuncture, part of the oriental medicine, has been used in eastern Asian countries for the management of various emotional, psychological, and psychiatric disorders including anxiety, stress, insomnia, and depression. In recent years, acupuncture has become one of the most popular complementary therapies in the West, and the therapeutic effectiveness of acupuncture on depression has been confirmed by modern research studies. Allen et al. [16] found that body acupuncture and auriculotherapy could significantly reduce the severity of depression. Similar results were demonstrated in the studies of Luo et al. [17] and Zhang et al. [18], in which researchers found that electroacupuncture was as efficacious as fluxetine in the management of major depression. In general, increasing evidences support that acupuncture is an effective treatment for patients with depressive disorders [19–23]. 

In the current study, we aimed to verify the vagus nerve responses during EA at ACR. Furthermore, antidepression effects of EA-ACR were investigated by observation of behaviors and measurement of blood biochemicals in the rat models of unpredictable chronic mild stress (UCMS). 

The results will provide a fundamental evidence for the anti-depression effects of EA-ACR and will facilitate EA-ACR to become a new noninvasive and low-cost therapy for depression.

## 2. Methods and Materials

### 2.1. Animals

Male Wistar rats in 150–170 g were obtained from the Laboratory Animal Resources Center, National Institute for the Control of Pharmaceutical and Biological Products, Beijing (Certificate no. SCXK (jing) 2009-0017). These animals were individually caged on a 12 h light/dark cycle (lights on at 8:00 a.m., lights off at 8:00 p.m.) under controlled temperature (22 ± 1°C) and humidity (50% ± 5%) conditions. Standard rat chow and water were given ad libitum. Animals were allowed to acclimatize for seven days before the study. All experiment procedures comply with the guidelines of the “Principles of Laboratory Animal Care” (NIH publication number 80-23, revised 1996) and the legislation of the People's Republic of China for the use and care of laboratory animals. The experimental protocols were approved by the Animal Experimentation Ethics Committee of the Institute of Acupuncture and Moxibustion, China Academy of Chinese Medical Sciences. Efforts were made to minimize the number of animal use and the suffering of the experimental animals.

### 2.2. Open Field Test for Behavioral Scoring

The open field apparatus was constructed of black plywood and measured 80 × 80 cm with 40 cm walls. White lines were drawn on the floor. The lines divided the floor into twenty-five 16 × 16 cm squares. A central square (16 cm × 16 cm) was drawn in the middle of the open field. Rats were put on the central square, at the same time the video camera was turned on for video recording from the top of the open field apparatus. Behaviors of rats were recorded for 3 minutes, with the grid number being counted as the horizontal score and the time of both frontal claws uplifting from the ground as the vertical score. The total locomotor activity of each animal was then scored as the sum of the number of line crosses and rears [24, 25]. 

### 2.3. Unpredictable Chronic Mild Stress (UCMS) Model

Eighty Rats were evenly randomized into 4 groups. Forty-two rats were recruited with the total score of 30–120 in the open field test [25]. A successful UCMS model rat was created with the score of the open field test equal or minus 60. Qualified rats were distributed into four groups: the normal control (*n* = 10), UCMS alone (*n* = 8), UCMS with EA-ACR treatment (EA-ACR) (*n* = 12), and UCMS with EA-ear-tip as the treatment control (EA-ear-tip) (*n* = 12). Every five rats in the normal group were housed in one cage. However, rats in the UCMS alone, EA-ACR, and EA-ear-tip groups were caged individually. Depression model was established by 21 days of UCMS combined with isolation. UCMS procedures were based on published studies [25, 26], including seven kinds of stressors: food deprivation, water deprivation, cage tilt 45° (Ugo Basile s.r.l. hot/cold plate, Model 35100–001, Italy), swimming in 4°C ice water, clipping tail 3 min, 50 V electric shock (Electronic stimulator, NIHON KOHDEN, Japan), and overnight illumination. The stressors were given randomly 3 times daily for 21 continuous days. The rats in the normal control group were housed undisturbedly except for necessary procedures such as routine cage cleaning.

### 2.4. Experimental Procedures (Figure 1)

**Figure 1:**
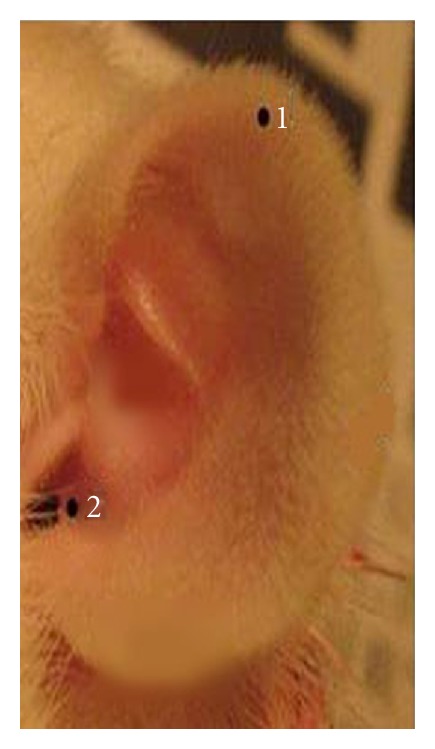
Electrical stimulation spot. Black spot 1 on the ear-tip area (nonauricular concha control). Black spot 2 on the auricular concha region (ACR). Positive iron pole (diameter 0.3 mm) on the frontal side of ear, and the negative iron pole (diameter 0.3 mm) on the back side.

The open field test on all rats was conducted on the day before the study, the 22th day (after UCMS), the 36th day (after treatment), and the 50th day in the study course. After the models of UCMS were established in 21 days, the EA treatment of 14 days was applied to the bilateral auricular concha region (Figure 1) of rats in the EA-ACR group once daily for 20 min. For the EA-ear-tip group, the EA applied to the bilateral ear tips (Figure 1) followed the same procedure and EA parameters as the EA-ACR. All rats in the EA groups accepted the inhaled anesthesia during the treatment. EA was set at the frequency of 2 Hz, the intensity of 1 mA by using the electroacupuncture stimulator (HANS-100A, Nanjing Gensun Medical Technology Co., Ltd., China). The inhaled anesthesia was conducted on the ISOFLURANE VAPORIZER (Matrx VIP 3000, Midmark corporation, USA) with isoflurane (Hebei Nine Sent Pharmaceutical Co., Ltd., Heibei, China). Blood pressure, including systolic, diastolic and mean pressures, and heart rate of rats were monitored noninvasively by using the apparatus (BP-98A, Beijing Soft Long Biological Technology Co., Ltd., China) during one EA treatment/anesthesia. The data were recorded in numerical values at the starting point of anesthesia (0 min pre-EA), the 1st min of anesthesia (EA begins), the 6th min of anesthesia (EA 5 min), the 11th min of anesthesia (EA 10 min), the 16th min of anesthesia (EA 15 min), and the 21st min of anesthesia (EA 20 min) on the same day for three UCMS groups. At the 51st day of the study, the rats were sacrificed and their neck venous blood was sampled for the tests of plasma cortisol (ELISA, R&D, USA) and ACTH (Acthlisa, R&D, USA) levels.

### 2.5. Statistical Analysis

The statistical analysis was performed by using one-way analysis of variance (ANOVA) followed by a Turkey test with software SPSS 13.0. *P* < 0.05 which was considered statistically significant, and the data were expressed as means ± standard deviation.

## 3. Results

### 3.1. Effects of EA-ACR Treatment on Heart Rate and Blood Pressure in UCMS Rats (Figures 2 and 3)

 No statistical difference was found in heart rate and blood pressure among rats of the three UCMS groups at the beginning of the study. Both of the heart rate and blood pressure in three UCMS groups showed a descending trend during the anesthesia period. However, the two EA treatment groups reduced heart rate and blood pressure significantly compared to the UCMS alone group. The mean heart rate from the 6th to 11th min decreased significantly in the EA-ACR group compared to the EA-ear-tip group; furthermore, the mean blood pressure was downregulated significantly in the EA-ACR group compared to the EA-ear-tip group in the treatment period. The EA-ACR treatment resulted in a significant decrease in the heart rate between the starting point of anesthesia and the 11th min of anesthesia (*P* < 0.05) and in the mean pressure between the starting point of anesthesia and the 16th min of anesthesia (*P* < 0.05). 

### 3.2. The Different Influences of EA-Treatments on the Open Field Test Score of UCMS Rats (Figure 4)

The total score of rats exposed to the open field test showed a significant decrease on the 22th day compared to the beginning in both the UCMS alone group and the EA-ear-tip group (*P* < 0.01 and *P* < 0.01, resp.). However, the score did not show remarkable differences between the two time spots in the normal group or in the EA-ACR group, respectively. But the EA-ACR group reached the score of 60 in the open field test and was thus qualified as the UCMS model. In the EA-ear-tip group, the score decreased significantly on the 36th day compared to the 22nd day. 

### 3.3. Effects of EA Treatment on Plasma Cortisol and ACTH Levels in UCMS Rats (Figures 5 and 6)

As compared with the normal group, plasma cortisol levels in the three UCMS groups showed significant increases (*P* < 0.01 for all comparisons). On the other hand, the mean plasma cortisol level of the EA-ACR group and the EA-ear-tip group decreased significantly compared to the UCMS alone group (*P* < 0.05 and *P* < 0.05, resp.). 

The 21-day UCMS exposure significantly increased the concentration of ACTH in rat blood (*P* < 0.01) when the UCMS group was compared with the normal control on the 51st day of the study. However, EA treatments significantly decreased the concentration of ACTH compared to the UCMS alone group (*P* < 0.01 and *P* < 0.01, resp.), while none of the EA treatment groups showed a significant difference in the concentration of ACTH as the normal control group did. 

## 4. Discussion

In general, EA treatments down-regulated the heart rate and blood pressure as well as the concentration of plasma cortisol and ACTH. However, the heart rate and blood pressure were influenced more intensively by the EA-ACR than the EA-ear-tip, and the open field test score was kept at a higher level by EA-ACR only.

### 4.1. Cardioinhibitory Effects of EA-ACR Are Similar to the Vagus Nerve Stimulation

 In the present study, EA-ACR elicited a significant decrease in heart rate and mean pressure under the anesthesia; however, the EA-ear-tip treatment did not induce similar changes during the treatment. These results suggested that EA stimulation at the auricular concha region induced similar effects as that of the direct vagus nerve electric stimulation had on the heart [7, 27, 28]. VNS, which stimulates the cervical trunk of the vagus nerve directly, is a procedure that was approved by FDA to treat primary hypertension years ago [7, 27]. Our previous research showed that acupuncture at auricular concha area could effectively decrease essential hypertension in rat models [9]. In addition, we found that electric stimulation on nucleus dorsalis nerve vagi could induce immediate decrease in heart rate and corresponding changes of electrocardiogram [29]. 

Anatomical knowledge of the vagus nerve informs that the auricular fibers of vagus nerve densely distribute in the concha and external auditory meatus of the ear; however, there are a few vagus nerve fibers around the ear tip [30]. The auricular branch of the vagus nerve ascends to the superior vagal ganglion (nucleus dorsalis nerve vagi), where the cholinergic preganglionic parasympathetic neurons give rise to the branchialefferentmotor fibers innervating the heart, and stimulating pathway induces cardioinhibitory effects [28]. Afferent signals elicited by EA-ACR may be integrated at the medulla oblongata, which then generates regulatory signals to activate the cardiac vagus nerve. Cardiac vagus nerve activation slows the heart rate and decreases the blood pressure immediately. 

### 4.2. EA-ACR Treatment Improved the Depressive Status of UCMS Rats in the Open Field Test

The unpredictable chronic mild stress (UCMS) has already contributed to the elucidation of the pathophysiological mechanisms of depression such as decreased neurogenesis and HPA axis alterations [26, 31]. In the current study, this model was used to explore the relations between depressive-like behavior in rats and EA-ACR treatment. The open field test provides simultaneous measurement of UCMS. A higher score in the test indicates increased locomotion and exploration and/or a lower level of anxiety [25, 26, 31]. In our study, the scores of open field test were significantly decreased in the UCMS alone and EA-ear-tip groups on day 22 compared to the beginning date, but no significant decrease was found in the EA-ACR group. However, all the rats in the EA-ACR group were qualified for modeling with a standard recruiting score of 60. It was apparent that EA-ACR kept the score on a higher level in the treatment course, while the score of the EA-ear-tip group showed a significant decrease during the treatment. This phenomenon indicated that EA-ACR induced the antidepressive effects. The UCMS model and the open field test were also successfully introduced into previous EA studies on depression. For example, EA at Baihui (GV20) and Yingtang (EX-HN3) on the top and front head scalp for 21 days can significantly improve the symptom of the depressive rats, the crossing and rearing movement times, and the number of p-CREB-positive neuron in the hippoeampus as the fluoxetine compared with the UCMS alone group [25]. 

### 4.3. EA Treatment Normalized the Hyperactivity of Hypothalamic-Pituitary-Adrenal (HPA) Axis

In the present study, 21 days' UCMS exposure significantly increased the concentrations of plasma cortisol and ACTH in rats. It is consistent with the previous research studies on both human beings and animal models with depressive status [32, 33]. Furthermore, 14 days of EA treatments (both EA-ACR and EA-ear-tip) right after UCMS down-regulated the plasma cortisol and ACTH in UCMS rats to normal levels. Researchers found that EA at acupoints Neiguan(PC6), Sanyinjiao(SP6), and Taichong(LV3) can lower plasma cortisol and ACTH levels and improve symptoms in depression [33].

Several hypotheses have been proposed for the pathological mechanism of depression. Besides disturbed monoaminergic neurotransmission, hyperactivity of hypothalamic-pituitary-adrenal (HPA) axis is closely related to major depression [34–36]. The HPA axis is the primary neuroendocrine system responsible for coordinating the mammalian stress response and has thus been a major focus of neurobiological research of depression. Major components of the HPA axis include corticotropin-releasing factor (CRF), adrenocorticotropin hormone (ACTH), and glucocorticoids. Cortisol is the major glucocorticoid in humans and animals. During stress response, neurons in the paraventricular nucleus (PVN) of the hypothalamus release CRF into the hypothalamo-pituitary portal system. CRF then stimulates the release of adrenocorticotropin (ACTH) from the anterior pituitary into systemic circulation, which in turn stimulates the adrenal cortex to secrete cortisol. Cortisol is responsible for many of the physiological changes associated with the stress response, and it also provides negative feedback to the hypothalamus and pituitary to decrease the synthesis and release of CRF and ACTH.

Patients with depression show hyperactivity of the HPA axis that may result from the impaired negative feedback regulation of glucocorticoid release [34]. Moreover, research study also found that normalization of these HPA axis abnormalities is associated with successful antidepressant treatment, and patients whose HPA abnormalities do not normalize are significantly more likely to relapse [37]. In a VNS treatment study, O'Keane et al. [38] found that the CRF/ACTH (adrenocorticotropic hormone) responses in the depressed group before VNS implantation were significantly higher than in the healthy group and were reduced to normal values after 3 months of VNS treatment; in addition, they also found significant improvement in depression symptoms. 

The result of the present study—EA-ACR significantly antagonized UCMS-induced depressive status of rats—is consistent with the findings of the mentioned research studies. As demonstrated through changes in plasma cortisol and ACTH levels, the antidepressant effect of EA-ACR may be mediated via normalization of the HPA axis hyperactivity. Otherwise, EA-ear-tip also was found to be the apparent down-regulation effect on the plasma cortisol and ACTH. It is found that a few vagus nerve fibers are around the ear tip [30], and HPA may be modulated by other nervous pathways beside the vagus nerve, for example, greater auricular nerve and lesser occipital nerve are densely innervated in the area of ear tip, and the EA signals can be transmitted by them to the cervical spinal cord and brain then modulate the HPA. Further investigation has been warranted for this hypothesis.

## 5. Limitation

This pilot EA-ACR study on depression has a small sample size. Meanwhile, EA-ACR does not only stimulate the vagus nerve, but also affects other sensory nerves, such as nervous auricularis magnus, lesser occipital nerve, facial nerve, and glossopharyngeal nerve fibers. Although EA-ACR elicited similar effects to VNS, the interaction among the nerves in the area should be explored in the future. Further investigation on EA-ACR for the disturbed monoaminergic neurotransmission of depression has been warranted.

## 6. Conclusions

EA-ACR can elicit similar cardioinhibitory effects to vagus nerve stimulation (VNS), and EA-ACR significantly antagonized UCMS-induced depressive status of rats. The antidepressant effect of EA-ACR is possibly mediated via normalization of the HPA axis hyperactivity. 

## Figures and Tables

**Figure 2:**
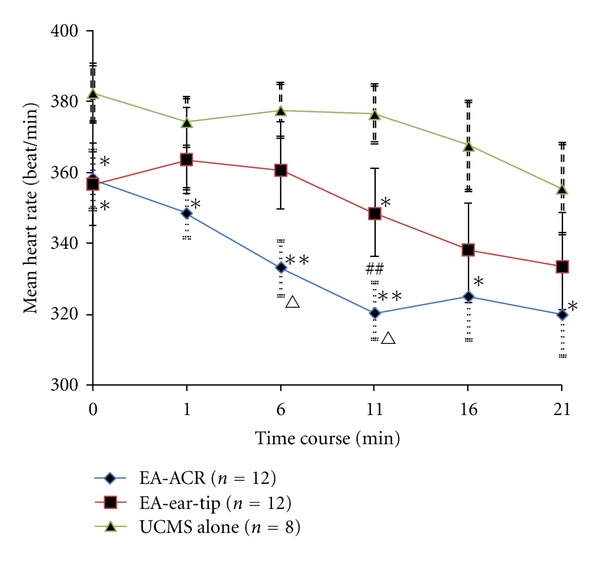
The time course of heart rate for three UCMS groups during one EA treatment/anesthesia. Comparison between the EA-treated UCMS group and the UCMS alone group, *P < 0.05, **P < 0.01. Comparison between the EA-ACR group and the EA-ear-tip group, ^∆^ *P* < 0.05, ^∆∆^ *P* < 0.01. Comparison between the starting point of anesthesia and the 11th min in the EA-ACR group. ^##^ *P* < 0.01.

**Figure 3:**
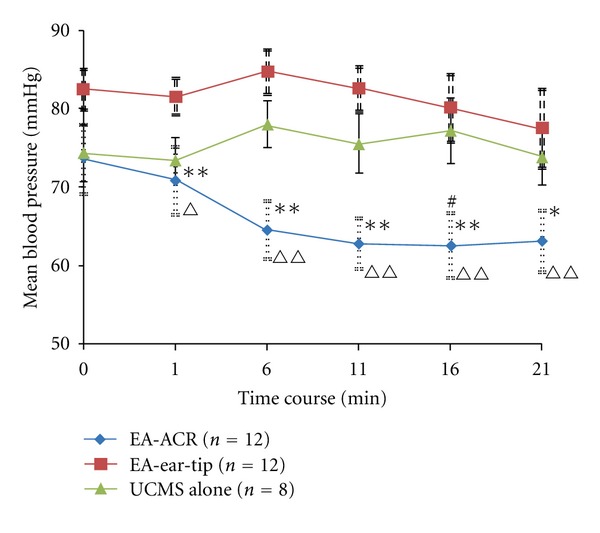
The time course of the mean blood pressure for the three UCMS groups during one EA treatment/anesthesia. Comparison between the EA-treated UCMS group and the UCMS alone group, *P < 0.05, **P < 0.01. Comparison between the EA-ACR group and the EA-ear-tip group, ^∆^ *P* < 0.05, ^∆∆^ *P* < 0.01. Comparison between the starting point of anesthesia and the 16th min in the EA-ACR group. ^#^ *P* < 0.05.

**Figure 4:**
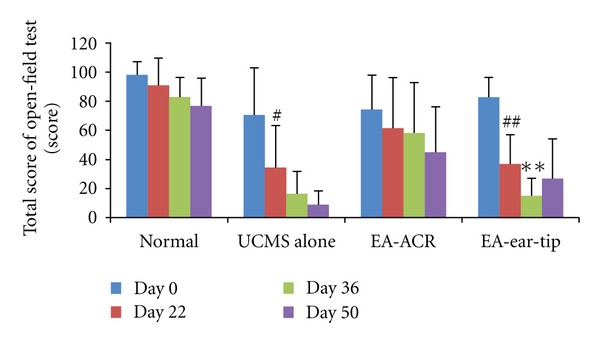
The influence of EA-ACR on the open field test score of rats. The 22nd day compared with the day before the test (day 0) in the same group, ^#^ *P* < 0.05, ^##^ *P* < 0.01; the 36th day compared with the 22nd day in the EA-ear-tip group, **P < 0.01.

**Figure 5:**
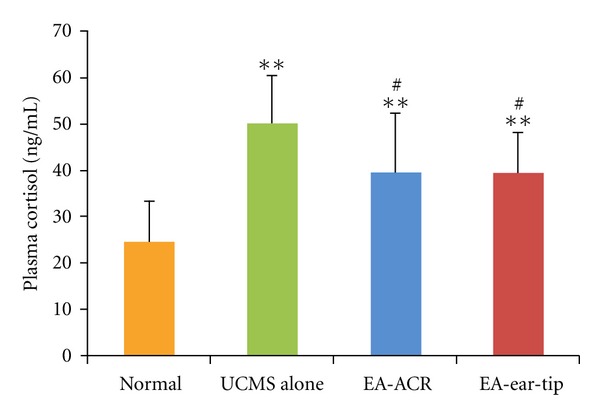
The effect of EA-ACR treatment on plasma cortisol in normal and UCMS rats. Comparison of plasma cortisol level between UCMS and normal groups, **P < 0.01. Comparison between the EA-treated UCMS group and the UCMS alone group, ^#^ *P* < 0.05.

**Figure 6:**
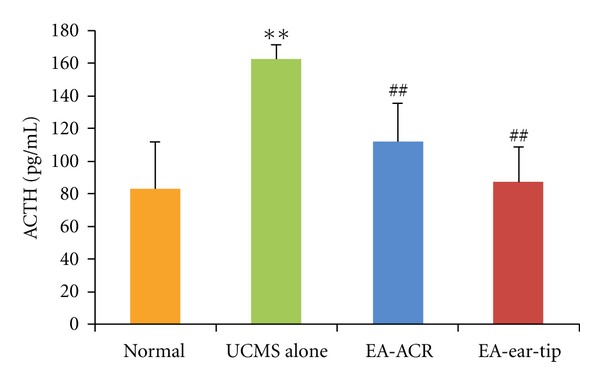
The effect of EA-ACR treatment on ACTH in normal and UCMS rats. Comparison of the ACTH level between normal and UCMS groups, **P < 0.01. Comparison between the EA-treated UCMS group and the UCMS alone group, ^##^ *P* < 0.01.
